# Sentinel surveillance system for early outbreak detection in Madagascar

**DOI:** 10.1186/1471-2458-10-31

**Published:** 2010-01-21

**Authors:** Laurence Randrianasolo, Yolande Raoelina, Maherisoa Ratsitorahina, Lisette Ravolomanana, Soa Andriamandimby, Jean-Michel Heraud, Fanjasoa Rakotomanana, Robinson Ramanjato, Armand Eugène Randrianarivo-Solofoniaina, Vincent Richard

**Affiliations:** 1 Unité d'Epidémiologie, Institut Pasteur de Madagascar, Antananarivo, République de Madagascar; 2 Service de la Lutte contre les Maladies Emergentes et Réémergentes, Direction des Urgences et de la Lutte contre les Maladies Transmissibles, Ministère de la Santé, du Planning Familial et de la Protection Sociale, Antananarivo, République de Madagascar; 3 Service de la Surveillance Epidémiologique, Direction des Urgences et de la Lutte contre les Maladies Transmissibles, Ministère de la Santé, du Planning Familial et de la Protection Sociale, Antananarivo, République de Madagascar; 4 Unité de Virologie, Institut Pasteur de Madagascar, Antananarivo, République de Madagascar

## Abstract

**Background:**

Following the outbreak of chikungunya in the Indian Ocean, the Ministry of Health directed the necessary development of an early outbreak detection system. A disease surveillance team including the Institut Pasteur in Madagascar (IPM) was organized to establish a sentinel syndromic-based surveillance system. The system, which was set up in March 2007, transmits patient data on a daily basis from the various voluntary general practitioners throughout the six provinces of the country to the IPM. We describe the challenges and steps involved in developing a sentinel surveillance system and the well-timed information it provides for improving public health decision-making.

**Methods:**

Surveillance was based on data collected from sentinel general practitioners (SGP). The SGPs report the sex, age, visit date and time, and symptoms of each new patient weekly, using forms addressed to the management team. However, the system is original in that SGPs also report data at least once a day, from Monday to Friday (number of fever cases, rapid test confirmed malaria, influenza, arboviral syndromes or diarrhoeal disease), by cellular telephone (encrypted message SMS). Information can also be validated by the management team, by mobile phone. This data transmission costs 120 ariary per day, less than US$1 per month.

**Results:**

In 2008, the sentinel surveillance system included 13 health centers, and identified 5 outbreaks. Of the 218,849 visits to SGPs, 12.2% were related to fever syndromes. Of these 26,669 fever cases, 12.3% were related to Dengue-like fever, 11.1% to Influenza-like illness and 9.7% to malaria cases confirmed by a specific rapid diagnostic test.

**Conclusion:**

The sentinel surveillance system represents the first nationwide real-time-like surveillance system ever established in Madagascar. Our findings should encourage other African countries to develop their own syndromic surveillance systems.

Prompt detection of an outbreak of infectious disease may lead to control measures that limit its impact and help prevent future outbreaks.

## Background

With the increase in international travel, infectious disease control is gaining a greater importance across regional borders [1]. Adequate surveillance systems are crucial for preventing the global spread of infectious disease [2]. Real-time syndrome-based surveillance provides the quickest way of identifying several diseases; thus, it is the best way of focusing the appropriate response measures to any outbreak scenario and to help identify specific etiologies [3].

Until March 2007, Malagasy surveillance system was only based on passive approach. Each primary health center reported monthly activities and diseases on a simple paper form. In the absence of diagnostic laboratories in most regions, the ability to confirm diagnosis remains low. Likewise, outbreaks must be notified using a forward stepwise approach involving each health system level, but that approach tends to be too slow for an adequate and efficient response. In fact, healthcare information systems have been driven mainly by the need to report aggregate statistics to Ministry of Health (MoH).

In March 2007, following the Indian Ocean chikungunya outbreak [4,5], which highlighted the absence of an efficient surveillance system in Madagascar and drew attention to the necessary improvement of systems for the early detection of outbreaks, the Malagasy MoH, with the support of the Institut Pasteur in Madagascar (IPM), set up a sentinel surveillance system based on daily data collection. The aim was to allow the rapid detection of an epidemic and to identify circulating arboviruses.

In general, viral surveillance does not allow normal viral circulation to be distinguished from a potential epidemic situation. Actually, the essential elements needed to plan for the prevention and control of epidemics cannot be obtained from a system based on virus surveillance alone, but requires a system based on both viral and clinical surveillance.

Prompt recognition of disease outbreaks and rapid diagnosis may permit prophylactic measures being introduced to decrease morbidity and mortality. However, the window of opportunity is short. This highlights the need for a rapid surveillance system that does not depend on confirmed diagnoses, but rather the detection of aberrant patterns. In focusing the appropriate outbreak response measures and helping to identify specific etiologies, syndrome-based real-time surveillance provides the quickest way of recognizing and responding to several disease outbreak scenarios [6-8].

The sentinel surveillance system in Madagascar was initiated to provide information using health service-based indicators and to alert health officials to changes in fever frequency during fever-associated diagnoses and changes in diarrheal frequency.

We aimed to describe the essential components of the Madagascar sentinel surveillance information system, and its results after one year.

## Methods

The surveillance uses health service-based indicators, and mostly focuses on fever syndromes. Since January 1, 2008, diarrhea has also been included as an indicator. Four sentinel primary health centers located in coastal cities with high population densities were also implemented with arbovirus surveillance.

### Management team

The first step in creating the sentinel fever network was to identify the appropriate stakeholders. The development team within the MoH and the IPM consisted of epidemiologists, virologists and managers of SSUREPI (epidemiological surveillance service, MoH) and SLMER (the service against emerging and re-emerging diseases, MoH). Next, immediate external stakeholders, including the regional health department and district managers, were brought into the discussion to identify potential sentinel centers.

### Sentinel primary health care centers

**Figure 1:**
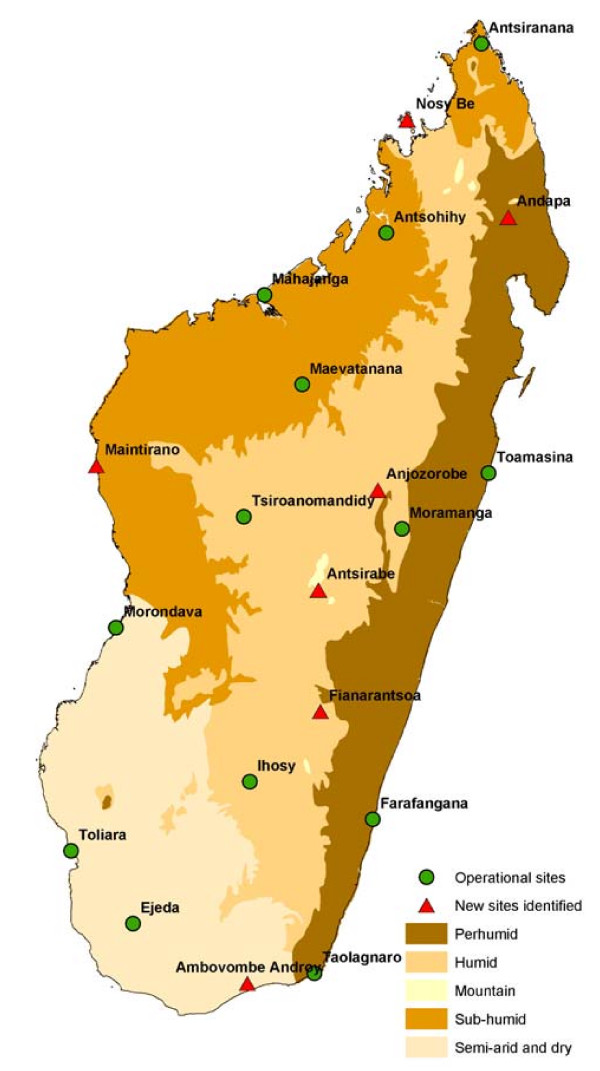
**Surrounding Climate and Location of the Health Centers that participated in the Sentinel Surveillance System in Madagascar**.

As Madagascar is a large island and have different climate patterns, implementation of the sentinel centers were carried to give geographical representativity and to provide best information about the trends in each of these climate areas. A sentinel center was selected based on several criteria, including the number of general practitioners (at last two by center), care level (activities, number of people that visit the center, equipments), communication facilities (mobile phone network availability) and motivation (voluntary participation) for the surveillance activity. The sentinel general practitioners, recruited on a voluntary and unpaid basis, are the backbone of this system. The centers numbered 13 on December 2008 and may increase to 20 over the next few months (figure 1).

### Data Collection

Surveillance was based on data collected from sentinel general practitioners (SGP). Each participating SGP reported the number of cases that met the criteria, including the total number of patient visits on each reporting day. SGPs were expected to communicate encrypted data via cellular telephone (encrypted SMS) at least once a day, from Monday to Friday, despite occasional clinic closures resulting from routine weekday-weekend schedules. If data from a sentinel center was not received by 08:00 a.m., the IPM staff member contacted the sentinel center to obtain the missing data.

The cost of data transmission was 120 ariary per day, less than 1 $ per month. Considering the lack of effective communication systems and money in Madagascar, the management team had to devise an efficient and cheap means of reporting data on a daily basis.

### Case definition

The sentinel surveillance system in Madagascar was based on clinical pre-diagnostic data. Fever (criteria for inclusion were an axillary temperature of more than 37.5°Celsius) was the first symptom targeted. Three illnesses in relation with fever were selected for surveillance: malaria confirmed cases (criteria for inclusion were fever with a positive result in the rapid diagnostic test); influenza-like illness (defined as fever with cough and sore throat); arbovirus (criteria for inclusion were fever without respiratory symptom and at least two other symptoms: headache, arthralgia, myalgia-like backache, skin rash, retro-orbital pain, haemorrhagic manifestations). Diarrheal disease was defined as 3 or more abnormally loose stools during the previous 48 hours. For each fever syndrome, a malaria rapid diagnostic test have to be performed using the CareStart™ Malaria kit provided by the malaria national program.

Furthermore, SGPs reported the sex, age, date and time of visit, and listed the chief complaint or symptoms for each new case via a paper form addressed to the IPM on a weekly basis. IPM staff were able to validate any information via mobile phone. However the notification was anonymous. The exchange between SGPs and IPM staff used only forms identification number. Data collection on a daily basis began in March 2007 and collection is ongoing. In terms of real-time surveillance, we are increasing the data entry and data transfer speed, with the aim of receiving most of the data within a 24-hour period. The data obtained daily from the SMS were entered into the Access^® ^database.

### Data analysis

Data were available for analysis shortly after the patient's initial visit. Data from all 13 primary health centers were evaluated for variation on a daily basis. A medical epidemiologist reviewed the output on a daily basis, and noted any changes to community-wide incidences of fever. Separate analyses were carried out for each syndrome category of interest, to look for temporal distribution increases at each sentinel center.

We estimated the proportion of all fever cases corresponding to each febrile syndrome at each sentinel center; we also estimated the proportion of all fever or diarrhea cases that were primary case encounters.

So, various health service-based indicators were monitored daily; these included the percentage of fever cases, confirmed malaria cases, influenza-like illness cases, arbovirus suspected cases, and the number of cases with diarrhea and diarrhea with fever.

**Figure 2:**
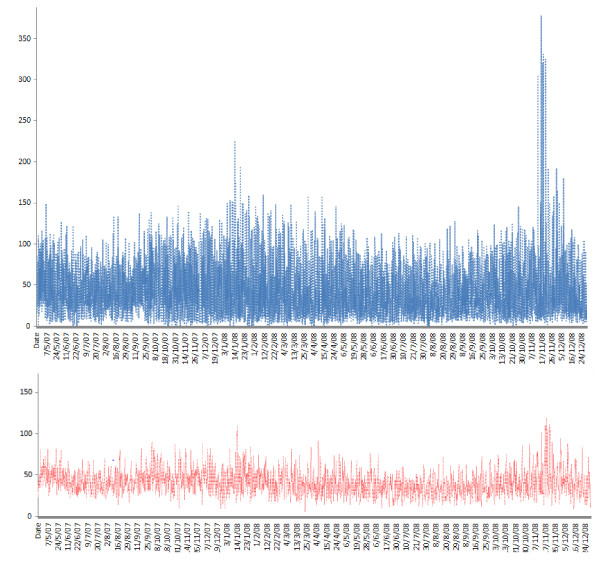
**Daily visit counts in the sentinel surveillance system in Madagascar and the moving average (over 5 days) for daily visit counts, April 10, 2007 - December 31, 2008**.

Surveillance data analysis was descriptive and straightforward using standard epidemiological techniques. Data were presented in the form of tables and graphs, and included the number of cases relating to each event (figure 2). Data were organized using statistical programs, which analyzed the daily and weekly graph values of various indicators, thus obtaining a baseline pattern for each syndrome in Madagascar.

Today, fluctuations in values in a sentinel center can be monitored on a daily basis, and increases can be detected through data analysis. Increases are reported immediately by telephone to SLMER and SSUREPI, to monitor events and to decide on whether to initiate an outbreak investigation.

### Ability to detect outbreaks

The sentinel surveillance system was able to detect peaks in the plots relating to febrile syndromes, including which disease among arbovirosis, influenza and malaria was the most probable cause. Without baseline levels, fluctuations were monitored daily, but the identification of aberrations was empirical and based on peak detection alone. Each increase was reported immediately by phone to regional health officials, and public health officials at the Ministry of Health. These district managers assisted with the interpretation and follow-up of aberrations in their perspective regions.

Further investigations were carried out if a change was detected. The first step in any outbreak investigation is to verify the signals, as the use of text message strings, to identify affected patients, may result in inclusion of patients whose main medical complaints are unrelated.

### Ethical clearance

The study was approved by the Ministry of Health and the National Ethics Committee of Madagascar.

## Results

### Implementation of the sentinel surveillance network

The surveillance network consisted of 13 centers in December 2008, which will increase to 20 by the end of 2009 (figure 1). During the planning phase, the advantages of sentinel surveillance were explained not only to public health officials but also to medical staff at participating health centers. Public health officials tended to prefer a weekly surveillance system, but daily transmission was easier for SGPs. More than three health centers in each sentinel town were contacted before the best center was chosen.

### Overview of the data - characteristics of the data on sentinel visits in Madagascar

Data, collected on a daily basis between April 1, 2007 and December 31, 2008 from the 13 sentinel centers and stored in the database, corresponded to 218,849 visits. Data was transmitted within 24 h in 89% of cases. The age distribution according to the overall number of visits and febrile syndromes are listed (Table 1).

**Table 1:** Distribution of overall visits and fever-related illnesses according to the age group.

Age group	All visits (n = 218,849)		Fever syndromes (n = 21,189)	
	n	%	n	%
<1 year	21,620	9.9	2,884	13.6
1-4 years	33,785	15.4	6,180	29.2
5-9 years	28,162	12.9	2,889	13.6
10-15 years	43,927	20.1	1,703	8.0
>15 years	91,355	41.7	7,533	35.5

A total of 26,669 cases (12.2%) presented a fever syndrome; a specific sheet was completed for 24,862 of these patients (93.2%). The sex ratio (male/female) for those with fever syndrome was 0.87. The age was available for 21,189 patients (85.2%), and the mean age was 14 years (CI95%: [13.8-14.2]).

Confirmed Malaria accounted for 9.7% of the overall number of fever-related illnesses, Flu-like syndromes accounted for 11.1% and Dengue-like syndromes accounted for 12.3%.

Understanding the characteristics and patterns of the number of visits over a period of time using this surveillance system in Madagascar was necessary before the appropriate threshold levels for various syndrome groups could be implemented for outbreak detection.

### Patterns of the Syndrome Groups

**Figure 3:**
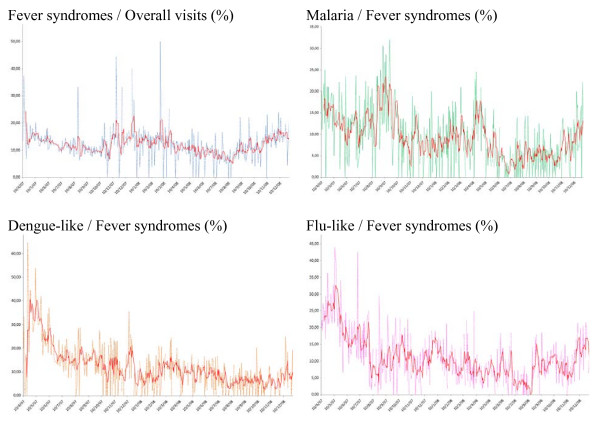
**Daily Sentinel surveillance time series plots (%) of total visits of fever and of total fever of the 3 syndrome groups and the moving average Apr. 10, 2007 - Dec. 31, 2008**.

To understand the epidemiological characteristics of groups with fever-related syndromes or those with rapid diagnostic test (RDT)-confirmed cases of malaria under the sentinel surveillance system, data of their daily counts were plotted on a graph (Figure 2 and 3, respectively). Daily and weekly counts as a function of the regional pattern were also plotted and analyzed for each sentinel center. Analysis of the weekly trends showed that the number of people visiting was greater on Monday (p < 0.001) than on any other day, probably related to the weekend closure effect. Figure 2 showed 2 peaks for daily visits count on January and November 2008 in relation with an increasing of fever syndrome on January (Figure 3).

The plots for global visits and fever-related visits were quite similar with a high correlation (R = 0.88). However, diarrheal syndrome was not highly correlated with global visits (R = 0.54).

Correlation between febrile syndrome and ILI syndrome (R = 0.73), febrile syndrome and dengue-like syndrome (R = 0.68) or febrile syndrome and confirmed cases of malaria (R = 0.72) was moderate.

**Figure 4:**
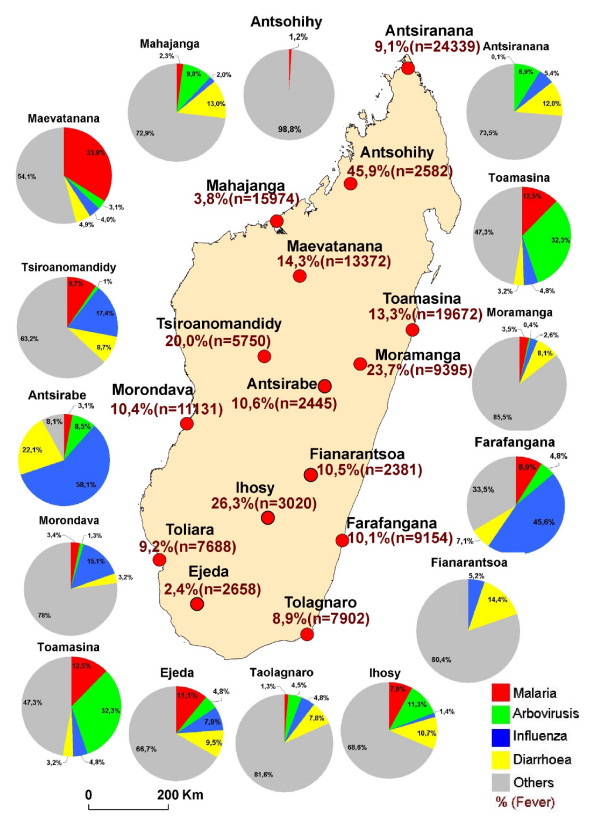
**Fever syndromes and proportions of fever-related syndromes under sentinel surveillance based on data collected from sentinel centers in 2008**.

The distribution of febrile and other syndromes in 2008 (figure 4) showed that arbovirosis was the dominant cause on the east coast, malaria was the dominant cause on the west coast and ILI was the dominant cause in a central strip between Morondava (West coast) and Farafangana (East coast).

### Outbreak detection

Ten cases of fever clusters occurred between March 2007 and December 2008, which led to an investigation after contact with the district managers. They were not detected by traditional surveillance system. Laboratory investigation confirmed the clinical signals and identified the etiological agents. The sentinel surveillance system confirmed five outbreaks: two via an increase in the dengue-like syndrome ratio without an increase in the fever-related ratio, in one of the four virological sentinel centers that have reported chikungunya virus circulation; two with an increase in fever and ILI indicators (Influenza A H1N1 seasonal); and one with an increase in RDT-confirmed cases of malaria.

## Discussion

Surveillance is fundamental to public health decision-making and subsequent action. Sustained and integrated global epidemiological surveillance has been weak in Madagascar, similar to that in other developing countries [2]. Sentinel surveillance systems offer advantages over passive surveillance, which is known to have limitations due to incomplete reporting [9]. The major objective of sentinel surveillance was to identify illness clusters early, before diagnoses are confirmed and reported to public health agencies, and to mobilize a rapid response, thereby reducing morbidity and mortality. To ensure efficiency and accuracy, sentinel systems require strong communication systems.

GPs participated in sentinel surveillance on a voluntary basis; therefore, the system is not representative of the whole country. To ensure better representation, the number of participating GPs should be increased. On the all 1500 health care centers directed by an physician, only 13 were included in the network. However, it was also important to ensure that sentinel GPs are easily accessible to surveillance staff. All sentinel centers were located in urban areas where population density was higher and where outbreak impact should be very dramatic. They also had been chosen to give an estimate picture of the different climate trend of Madagascar. The estimated population covered by all sentinel sites is 500.000 inhabitant representing as of 2008 3% of the Malagasy population.

Supervision of the surveillance system was carried out by MOH as planned. Stakeholders were made aware of the advantages and limitations of sentinel surveillance systems. Sentinel surveillance systems may enhance collaboration among health ministry services and health-care practitioners. However, they do not replace traditional public health surveillance, nor are they substitutes for direct medical analysis, in which the physician reports unusual or suspect cases of public health importance.

Establishing sentinel surveillance was a difficult process, even if it was less costly and used fewer resources than passive surveillance. The problem, among others, in establishing such a system involves connecting GPs to the sentinel system and coordinating their work. We selected mobile phone communication for daily reporting and paper forms for weekly reporting, increasing the human resources required to carry out this type of surveillance. In the future, with the increase in mobile communication in Madagascar, in particular that involving 3G internet, sentinel surveillance could be based on electronic records delivered directly from the practitioners.

Although the cost of data transmission on a daily basis is minimal, at less than 1US$ per month per sentinel center, the costs and maintenance of the system require better quantification, both in terms of resources spent (time of the SGPs,...) and person-hours involved in responding to system alerts. Furthermore, the importance of malaria in Madagascar and other sub-Saharan countries requires that a rapid malarial test be linked to fever syndrome surveillance.

The mobile phone with its low cost and universal availability has been recognized as an important piece of communication technology in health care [10]. However, published studies on using mobile phones are still limited [11] to countries where limited resources and remote locations are the main reasons for using mobile phone.

Sentinel surveillance systems seek to use existing health data in real time (daily) to provide immediate analysis and feedback to those charged with the investigation and follow-up of potential outbreaks. However, data relating to these various syndromes are nonspecific by nature. For illnesses that are self-limiting and of a short duration, resolving the causes of the syndrome is not usually of direct benefit to the patient, is not a priority for the clinician, and is not always feasible with current technology. The advantage of using syndromic data for outbreak detection is speed of response [12]. Experiences to date indicate that this advantage may only be theoretical. The time required to conduct investigations and retrieve diagnostic and epidemiological information might negate the advantage of quick data acquisition. The absence of sustained syndromic signals usually provides greater reassurance that an outbreak does not exist than the information obtained by immediate investigation.

The rapidity of the system, although excellent compared with traditional surveillance systems, needs to be improved. This Malagasy sentinel surveillance system did detected fever outbreaks in areas where traditional surveillance system didn't. It also highlighted the difficulty in building epidemiological baselines without historical references.

Systematic methods for determining the expected number of visits on a particular day require historical data to create baselines in trimmed-mean seasonal and autoregressive integrated moving average (ARIMA) models. Time series methods are an important tool to provide alarm thresholds. These forecasting models could produce results with good accuracy in their predictions of the data [13].

## Conclusion

A sentinel real-time-like surveillance system may be the key to the detection of and prompt reaction to any infectious disease outbreak. Although preliminary, the results showed the feasibility of implementing syndrome surveillance in a developing country at low cost, with good cooperation by SGPs (daily data transfer rate estimated to 89%) and a minimum of effort by staff. This is the first description of a sentinel surveillance system based on daily SMS reporting. We believe that this very simple system may provide an example to several other developing -- and even developed -- countries. Although the cost of data transmission was minimal, the cost and maintenance requirements of the system need to be better quantified, both in terms of resources spent and person-hours used to respond to system alerts. The time required to conduct investigations and retrieve diagnostic and epidemiological information might negate the advantage of timely data acquisition, especially in developing countries where the money for investigation can be difficult to find. For this reason, the Madagascar Ministry of Health has dedicated funds to outbreak investigation, in addition to those invested in the surveillance system.

The sentinel surveillance system is a key step in closing the gap that exists in the surveillance of disease in Madagascar. However efficient, this system cannot replace traditional surveillance, nor can it substitute for the direct reporting of unusual or suspect cases of public health importance by physicians. Epidemiological baselines for each center need to be determined, to help develop better statistical methods and sensible alarm thresholds, which can then be extended to more sentinel centers.

Our findings should encourage other African countries to develop functional, patient-based sentinel surveillance systems, while discovering their inherent advantages and limitations.

## Competing interests

The authors declare that they have no competing interests.

## Authors' contributions

RL was in charge of epidemiological data analysis, improvement of the Sentinel Surveillance System and manuscript writing. RY initiated the project for detecting outbreaks and participated in the task force. RL participated in the task force. ASF and HJM were in charge of biological surveillance. RM was in charge of epidemiological data analysis. RR and RSA participated in the task force. RV led the study and coordinated the data analysis manuscript writing, and participated in the task force.

All authors read and approved the final manuscript

## Pre-publication history

The pre-publication history for this paper can be accessed here:

http://www.biomedcentral.com/1471-2458/10/31/prepub
